# Effects of a personalised, adapted computerised cognitive stimulation programme versus stimulating leisure activities in younger and older adults with mild or subjective cognitive impairment. Protocol for a randomised controlled trial

**DOI:** 10.23938/ASSN.1118

**Published:** 2025-04-30

**Authors:** Isabel Gómez-Soria, Bárbara Oliván-Blázquez, Alejandra Aguilar-Latorre, Juan Nicolás Cuenca-Zaldívar, Rosa Mª Magallón-Botaya, Estela Calatayud

**Affiliations:** 1 University of Zaragoza Faculty of Health Sciences Department of Physiatry and Nursing Zaragoza Spain; 2 Institute for Health Research Aragón (IIS Aragón) Zaragoza Spain; 3 Aragones Group of Research in Primary Health Care (GAIAP) Zaragoza Spain; 4 University of Zaragoza Faculty of Social and Labor Sciences Department of Psychology and Sociology Zaragoza Spain; 5 University of Zaragoza Faculty of Human Sciences and Education of Huesca Department of Psychology and Sociology Huesca Spain; 6 Research Group in Nursing and Health Care Puerta de Hierro Health Research Institute - Segovia de Arana (IDIPHISA) Majadahonda Madrid Spain; 7 Primary Health Center El Abajon Las Rozas Madrid Spain; 8 Universidad de Alcalá Facultad de Medicina y Ciencias de la Salud. Departamento de Enfermería y Fisioterapia Grupo de Investigación en Fisioterapia y Dolor Alcalá de Henares Spain; 9 University of Zaragoza Faculty of Medicine Department of Medicine, Psychiatry and Dermatology Zaragoza Spain

**Keywords:** Mild cognitive impairment, Subjective cognitive impairment, Computerized cognitive stimulation, Stimulating leisure activities, Primary Care, Deterioro cognitivo leve, Deteriro cognitivo subjetivo, Estimulación cognitiva computarizada, Actividades de ocio estimulantes, Atención Primaria

## Abstract

**Background:**

Mild cognitive impairment represents a transitional stage between healthy aging and dementia, with subjective cognitive impairment being a key predictor of progression to dementia. This randomized controlled trial aims to compare the effectiveness of a personalized computerized cognitive stimulation program with that of stimulating leisure activities in younger and older adults with mild or subjective cognitive impairment.

**Methods:**

Participants aged ≥ 50 with mild cognitive impairment and subjective cognitive impairment or scores between 24 and 31 on the Spanish Mini-Mental State Examination were recruited. Exclusion criteria comprised living in residential care, use of acetylcholinesterase inhibitors, sensory impairments, agitation, or having received cognitive stimulation in the past 12 months. Fifty-nine community-dwelling individuals in Zaragoza, Spain, were randomly assigned to an two interventions group or a control group. The first intervention group will receive personalized computerized cognitive stimulation for 30 minutes per day, five days per week, while the second intervention group will participate in two to five stimulating leisure activities. The intervention will last eight weeks. The control group will receive the usual care for the same duration. The primary outcome is the assessment of global cognition; secondary outcomes include memory, verbal fluency, activities of daily living, and mood.

## RESUMEN

**Fundamento:**

El deterioro cognitivo leve representa una etapa de transición entre el envejecimiento saludable y la demencia; el deterioro cognitivo subjetivo es un predictor clave de esta progresión. Este ensayo controlado aleatorizado tiene como objetivo comparar la eficacia de un programa personalizado de estimulación cognitiva informatizada frente a la realización de actividades de ocio estimulantes en personas adultas jóvenes y mayores con deterioro cognitivo leve o deterioro cognitivo subjetivo.

**Métodos:**

Se reclutaron participantes ≥50 años con deterioro cognitivo leve, deterioro cognitivo subjetivo, o que obtuvieron puntuaciones de 24-31 puntos en el Mini-Examen Cognitivo de Lobo (versión de 35 puntos). Los criterios de exclusión incluyeron vivir en una residencia, tomar inhibidores de la acetilcolinesterasa, presentar alteraciones sensoriales, agitación o haber recibido estimulación cognitiva en los últimos 12 meses. Cincuenta y nueve personas residentes en la comunidad en Zaragoza, España, fueron asignadas aleatoriamente a dos grupos de intervención o al grupo control. El primer grupo de intervención recibirá estimulación cognitiva informatizada personalizada durante 30 minutos/día, 5 días/semana mientras el segundo grupo de intervención participará en dos a cinco actividades de ocio estimulantes. La intervención durará ocho semanas. El grupo control recibirá la atención habitual durante el mismo periodo de tiempo. El resultado primario corresponde a la evaluación de la cognición global; los resultados secundarios comprenden mediciones de memoria, fluidez verbal, funcionamiento en actividades de la vida diaria y estado afectivo.

## INTRODUCTION

The global prevalence of dementia is projected to rise from 57.4 million cases in 2019 to an estimated 152.8 million by 2050^1^. Alzheimer’s disease (AD) is the most common form of dementia and ranks among the leading causes of death worldwide^2^.

Current research guidelines describe six clinical stages of AD. Stage 3, which represents the prodromal stage of the disease and is commonly referred to as mild cognitive impairment (MCI), is the stage immediately preceding AD. Stage 2 marks an earlier transitional phase, characterized by subtle cognitive changes that do not yet fulfil the diagnostic criteria for MCI. This stage is referred to as subjective cognitive impairment (SCI)^3^.

SCI is considered an earlier indicator in the continuum between healthy cognitive ageing and MCI. It is defined as a self-perceived decline of cognitive functioning despite normal performance on standardized neurophysiological tests^4^. Evidence suggests that individuals with SCI are at increased risk of progressing to dementia (HR = 1.90, 95% CI 1.52-2.36; OR = 2.48, 95% CI 1.97-3.14) and MCI (HR = 1.73, 95% CI 1.18-2.52; OR = 1.83, 95% CI 1.56-2.16)^5^

Cognitive neuro-constructs commonly affected in individuals with SCI include memory language, executive functioning, visuospatial abilities, orientation, and attention^6^. Self-reported memory-specific difficulties are associated with long-term decline in global cognitive functioning over a six-year period and may serve as predictors of incident dementia, particularly in subjects experiencing depression and/or anxiety^7^.

MCI is widely recognized as an intermediate stage in the continuum between normal cognitive ageing and dementia^8^. The average annual conversion rate from MCI to dementia is approximately 12.2%, nearly three times higher than that observed in the general population^9^. The overall global prevalence of MCI is estimated at 15.56% (95% CI: 13.24-18.03%), with prevalence increasing from 10.88% (95% CI: 5.37-17.98%) in individuals aged 50-59 years to 21.27% (95% CI: 16.26-26.75%) in those aged ≥ 80^10^.

MCI can present in various subtypes depending on the nature and extent of cognitive deficits. These subtypes include single-domain amnestic, multi-domain amnestic, non-amnestic single-domain, and non-amnestic multi-domain)^11^^,^^12^. The classification depends on whether memory is the primary domain affected and whether other cognitive domains are also impaired.

In individuals with amnestic MCI (aMCI), greater difficulties are observed in tasks involving semantic fluency and verbal comprehension compared to those with non-amnestic (naMCI)^13^. Furthermore, the presence of frontal-executive dysfunction in aMCI is associated with a poorer prognosis, highlighting the need for prioritizing these individuals in intervention strategies^14^. Evidence also suggests that adults with naMCI may benefit from intense cognitive interventions, whereas those with the amnesic subtype may respond better to less intensive but more socially engaging activities^15^ The classification of MCI into distinct subtypes is valuable for differentiating the risk of progression to various forms of dementia. It also enables the identifications of individuals at high risk of developing dementia based on specific cognitive profile^16^.

The cognitive domains most commonly affected in MCI include memory, attention, executive functions, orientation, visuospatial skills, calculation, visual perception^17^, perceptual motor, social cognition^18^, social functioning, processing speed, learning, and semantic language^19^. Furthermore, impairment in memory, executive function, and processing speed have been associated with increased cognitive decline in individuals experiencing mood disorders such as depression and anxiety^20^.

Depressive symptoms are common in individuals with MCI, affecting approximately 32% of this population, while anxiety disorders occur in about 14%^21^. Anxiety symptoms have been shown to increase the risk of progression from SCI to objective cognitive impairment^22^, while both anxiety ^(^^23^ and depressive symptoms^24^ elevate the risk of progression from MCI to dementia.

Evidence indicates that some individuals with MCI remain cognitively stable for years^25^, suggesting that this condition may be treated with non-pharmacological interventions (NPI). These interventions include non-invasive, cost-effective, safe, and easy to implement approaches. Their effectiveness is significantly influenced by factors such as frequency, duration, mode of administration, and environmental context^26^. Among these, cognitive stimulation (CS) is possibly the best option^27^, while stimulating leisure activities (SLA) are widely utilized in occupational therapy (OT)^28^.

Occupational therapists play a crucial role within multidisciplinary primary care teams^29^, emphasizing the importance of engaging individuals in meaningful occupations to support mental, physical, and social health^30^.

CS is relevant for the OT, aligning with key principles, such as patient orientation, activity analysis, activity classification, and meaningful occupational participation^31^. It is defined as “an intervention involving *mental exercises* through enjoyable activities designed to stimulate thinking, concentration, and memory, which can be conducted in small groups or individually” ^32^.

CS has demonstrated the potential in improving global cognition among individuals with MCI^33^. These interventions are typically categorised based on their duration: *short-term* (less than three months); *maintenance or medium-term* (three to six months), and *long-term* (more than 12 months)^34^. Notably, maintenance CS has emerged as the non-pharmacological intervention with the strongest evidence of cost-effectiveness for managing MCI and dementia^35^. Primary care physicians often recommend CS for patients with SCI and MCI as a strategy to reduce cognitive decline and help prevent the progression of dementia^36^.

Leisure activities, defined as voluntary, non-work activities for enjoyment and reflecting personal interests, have been associated with a reduced risk of dementia in older adults^37^. These activities not only provide pleasure but also contribute to cognitive health and overall well-being^38^. Social prescribing, which enables personalized care for community-dwelling individuals^39^, aims to promote well-being and foster a sense of belonging^40^. Furthermore, leisure activities play a significant role in supporting the health improvement objectives of formal healthcare services^41^.

Rotenberg et al. 2022^42^, report that individuals with SCI and MCI often experience difficulties in social and leisure activities, as well as in time management. In this context, occupational therapists may recommend participation in meaningful activities to promote health and prevent cognitive decline^43^.

Previous studies have demonstrated the benefits of traditional personalized CS for individuals with MCI^44^^-^^47^ and SCI^47^^,^^48^. Some research has examined the effects of computerised CS in older adults with MCI^49^^-^^52^; however, these studies did not utilize personalised approaches. Other studies have evaluated the impact of mentally SLA on MCI^53^^-^^55^, but none have directly compared the effects of computerised CS versus SLA in both younger and older adults with MCI.

This study aims to evaluate the effectiveness of a personalised and adapted computerised CS programme (IG1) implemented in a primary care health centre versus SLA (IG2) in community-dwelling adults aged ≥ 50 years with MCI and SCI. The evaluation will focus on global cognition, memory, verbal fluency, ADLs, IADLs, symptoms of depression and anxiety.

## METHODS

### Study design

**Figure 1:**
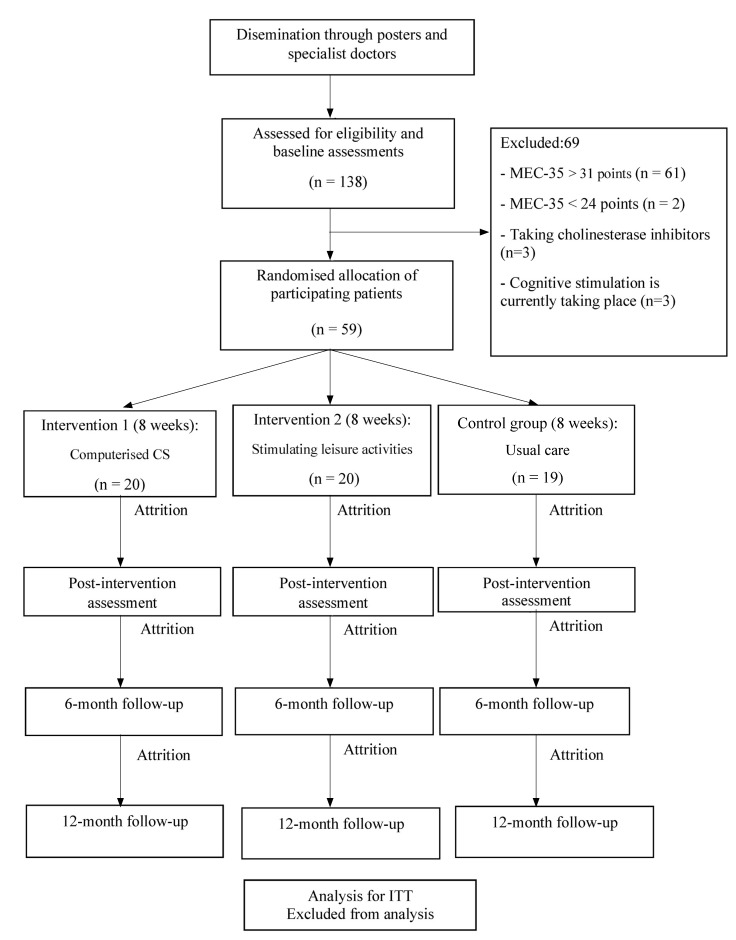
Protocol of the randomised controlled trial.

Randomized, controlled, single-blind trial, involving 59 participants that will be allocated into three parallel groups (Fig. 1): two intervention groups (IG1 and IG2) and a control group (CG).

### Participants

The study population comprised younger and older adults with possible MCI or SCI, recruited from primary care consultations across three health centres in El Rabal (Zaragoza, Spain). The aging index of the study area is 157% and the dementia rate is reported to be 9.7 cases per 1,000 inhabitants. The demographic distribution is approximately 49% males, with an average age of 43 years, and 51% female, averaging 45 years. The child population constitute 13% while the youth population account for 64% of the total^56^.

### Eligibility criteria (inclusion and exclusion criteria)

*Inclusion criteria*

- ≥ 50 years
- Living in the community
- Diagnosed with MCI or scoring between 24 and 27 points on the MEC-35, which may indicate the presence of MCI^57^.
- Scoring between 28 and 31 points on the MEC-35, which may suggest SCI^47^.

*Exclusion criteria*

- Living in residential homes.
- Taking acetylcholinesterase inhibitors, as they may act on global cognition and/or cognitive function.
- Significant sensory deficits that prevent the intervention from being conducted.
- Agitation, physical or verbal, with aggressive behaviour, which makes it difficult to conduct the interventions.
- Having received CS or memory workshops in the last 12 months.

### Recruitment and enrolment

Recruitment began with the placement of information posters in three health centres in Zaragoza (*Arrabal*, *Barrio Jesús*, and *La Jota*). Next, trained professionals - an occupational therapist and a psychologist - carried out the recruitment at each centre. These professionals provided detailed information about the study, including inclusion criteria, study objectives, and participation requirements based on group assignments. They also will distributed mini-posters listing contact information and office hours for enrolment. Interested individuals were invited to schedule appointment and will be informed of the date, time, and location. During this appointment, a psychologist will conducted cognitive screening using the MEC-35. Participants who score between 24 and 31 points on this assessment and meet the remaining eligibility criteria were invited to join the study. Each eligible participant received a comprehensive explanation of the study and an information sheet. Written informed consent were obtained before enrolment.

### Randomisation and blinding

The principal investigator confirmed eligibility and performed individual randomisation. Participants were allocated using a gender- and cognitive level-stratified random number sequence generated with R version 4.1.3 (R Foundation for Statistical Computing, Institute for Statistics and Mathematics, Welthandelsplatz 1, 1020 Vienna, Austria). Participants were randomly assigned in a 1:1 ratio to one of three groups regardless of their health centre: 20 participants in Intervention Group 1 (IG1), 20 in Intervention Group 2 (IG2), and 19 in the Control Group (CG). While participants were aware of their group assignment, outcome assessors were blinded. All assessors underwent theoretical and practical training and were distinct from those delivering the interventions. Two specialized occupational therapists conducted intervention sessions. Although participant blinding was not feasible, adherence to intervention protocols was monitored. Attendance and compliance with assigned activities were recorded in a field diary.

### Intervention procedures

#### Computerized cognitive stimulation

The intervention will be delivered by an occupational therapist through a combination of in-person sessions and home-based computerized CS.

The therapist will conduct three face-to-face sessions in small groups, each lasting between 60 and 90 minutes. During these sessions, participants will be instructed to check and mark the calendar daily to enhance temporal-spatial orientation. Guidance will be provided on how to optimize the training environment - such as ensuring adequate lighting and performing exercises in the morning -. Additionally, participants will be trained in the use of the CS platform and will be engaged in group activities as examples of the types of exercises included.

In the first face-to face session, a health education talk will be delivered, focused on protective factors against cognitive decline and offering practical recommendations.

Throughout the intervention, participants will receive the following cognitive and lifestyle advice via the CS platform:

- Write down important weekly reminders on a whiteboard
- Follow a Mediterranean diet
- Make a shopping list before going to the store
- Aim for 8 hours of sleep and rest.
- Perform exercises in a quiet, well-lit room
- Record medical appointments on a calendar.
- Strengthen memory by memorizing phone numbers of close contacts.
- Recall and share popular sayings with others.

In addition, participants in IG1 will receive specific training on how to use the computerised CS platform *Stimulus*, along with a short talk explaining key cognitive neuroconstructs targeted during the intervention and relevant advice.

At home, IG1 participants will engage in personalised and adapted computerised CS using the *Stimulus* platform for 30 minutes per day, 5 days per week, over a period of 8 weeks (totalling 40 sessions).

The duration of the interventions will be based on several considerations:

- Cognitive functions, particularly attention, often decline with age^58^, and attentional deficits are common in MCI^59^;
- Woods et al. 2023^32^ suggest that CS sessions may range from 30 to 120 minutes, with frequencies of one to six times per week;
- Leroi et al. 2019^60^ applied 30-minute CS sessions in individuals with Parkinson’s disease and MCI;
- Evidence from an 8-week traditional CS intervention showed cognitive benefits^44^.

The CS program targeted multiple cognitive domains, including short-term memory, long-term memory, language, calculation, perception, logical reasoning, attention, executive functions, processing speed, and visual-motor skills. The use of external memory aids was encouraged.

The computerised CS intervention will be adapted according to the participant’s cognitive level, as assessed by the MEC-35, and graded in difficulty to allow for progression or regression based on individual performance over time.

Tables present key details of the intervention. Table 1 outlines the cognitive aspects (neuroconstructs), objectives, and types of computerised CS exercises used, while Table 2 shows the weekly intervention plan for participants with MCI and with SCI.

**Table 1:** Neuroconstructs, aims and types of computerised CS exercises used in intervention

Neuro-constructs / Objectives	Type of computerised cognitive stimulation exercises
*Short-term memory*	
- Compensate for memory lapses through different strategies and resources.	- Remembering words
	- Remembering objects and their names
	- Remembering the last letters
	- Repeating the sequence of colours
	- Remembering the last letters
	- Remembering the last position of the square on the board
	- Remembering the illuminated squares
	- Play the spatial sequence
	- Remembering the key
	- Matching faces and names
	- Paint the figure
	- Remembering the squares on a symmetrical board
*Attention*	
- Stimulate attention associated with memory and the perception associated with it.	- Letter recognition
- To promote voluntary, selective and sustained attention.	- Recognising numbers
	- Recognising geometric shapes
	- Recognising colours
	- Finding words
	- Determine the position area of the object
	- Determine if the word was shown previously
	- Determine if the object was shown previously
	- Stimulate attention associated with memory and the perception associated with it.
	- To promote voluntary, selective and sustained attention.
*Calculation*	
- To exercise arithmetic skills: addition, subtraction and multiplication.	- Solve operations
- Solving mathematical problems.	- Order numbers
	- Calculate the amount
	- Indicate the result of the operation
	- Fill in the missing number
*Language*	
- Sort words to form a sentence.	- Stimulate verbal comprehension and verbal expression.
- Decide if it belongs to a category.	- To exercise automatic language.
- Identify which letter it starts with.	- To preserve and/or strengthen reading and writing skills.
	- To stimulate verbal fluency.
*Processing speed*	
- Improve responsiveness in performing different activities, in decision making and in planning activities of daily living.	- Detect objects while driving
	- Track last location
	- Find copy
*Executive functions*	
- Exercise planning skills.	- Order sequences of an action.
	- Follow two balls.
	- Remembering the triangle.
	- Train task switching.
	- Identify intruder.
*Long-term memory*	
- Maintaining long-term memory.	- Finding a partner.
	- Playing instruments.
*Reasoning*	
- To stimulate the capacity for abstraction.	- Solve series of letters.
	- Calculate bus departure time.
	- Point out repeated letters in a series.
	- Identify the different series.
	- Marking pattern changes.
	- Point out repeated objects in a series.
	- Point out repeated words in a series.
	- Detect the discordant element.
	- Solve series of figures.
*Perception*	
- To promote visual-spatial organisation.	- Divide into two equal parts.
- To interpret the reality of time.	- Estimate time.
	- Locate the triangles equal to the model.
*Visual motor skills*	
- Follow the ball	- Improve hand-eye coordination as we will direct our attention to the visual stimulus and with our hands we will carry out the tasks and activities.
- Join the dots	- To promote the performance of activities and tasks in which the eyes and hands are used simultaneously, with a consequent improvement in the performance of ADLs.
- Copy the model	

**Table 2:** Weekly schedule of cognitive stimulation for participants with mild (MCI) and severe cognitive impairment (SCI)

Week	Participants with MCI	Participants with SCI
1	Working memory	Working memory
	Long-term memory	Long-term memory
	Attention	Attention
	Language	Language
	Calculation	Executive functions
	Executive functions	Processing speed
	Perception	Visual-motor skills
	Reasoning	Perception
	Processing speed	
2	Working memory	Working memory
	Attention	Attention
	Language	Language
	Calculation	Executive functions
	Processing speed	Reasoning
	Perception	Calculation
	Visual-motor skills	Processing speed
	Executive functions	Visual-motor skills
3	Working memory	Working memory
	Long-term memory	Long-term memory
	Attention	Attention
	Language	Language
	Calculation	Executive functions
	Executive functions	Visual-motor skills
	Perception	Perception
	Reasoning	Calculation
	Processing speed	
4	Working memory	Working memory
	Attention	Attention
	Language	Language
	Calculation	Executive functions
	Processing speed	Reasoning
	Perception	Calculation
	Visual-motor skills	Processing speed
	Executive functions	Visual-motor skills
5	Working memory	Working memory
	Long-term memory	Long-term memory
	Attention	Attention
	Language	Language
	Calculation	Executive functions
	Processing speed	Processing speed
	Perception	Visual-motor skills
	Visual-motor skills	Perception
	Executive functions	
6	Working memory	Working memory
	Long-term memory	Attention
	Attention	Language
	Language	Executive functions
	Calculation	Reasoning
	Executive functions	Calculation
	Perception	Processing speed
	Reasoning	Visual-motor skills
	Processing speed	
7	Working memory	Working memory
	Attention	Long-term memory
	Language	Attention
	Calculation	Language
	Processing speed	Executive functions
	Perception	Visual-motor skills
	Visual-motor skills	Perception
	Executive functions	Calculation
8	Working memory	Working memory
	Long-term memory	Attention
	Attention	Language
	Language	Executive functions
	Calculation	Reasoning
	Executive functions	Calculation
	Perception	Processing speed
	Reasoning	Visual-motor skills
	Processing speed	

#### Stimulating leisure activities

Participants in IG2 will take part in three face-to-face group sessions lasting between 60 and 90 minutes in small groups. In the first session, participants will attend an educational talk introducing the variety of cognitively stimulating leisure activities. The benefits of engaging in cognitive, physical, and social leisure activities will be explained, as well as guidance on how to complete the weekly questionnaire. In the subsequent sessions, any questions will be clarified.

Over the course of eight weeks, IG2 participants will perform two to five cognitively stimulating leisure activities per week. These activities will be selected from an adapted version of Karp et al. 2006^61^. Each week, IG2 participants will indicate which cognitively stimulating activity they performed along with the daily frequency (less than 30 minutes, 30 minutes to 1 to hour, 1 to 2 hours, or more than 2 hours).

#### Control group

Participants in the CG will not receive either of the two interventions (CS or SLA) during the study period. However, they will attend a face-to-face session lasting 90 minutes, during which they will receive a health education talk focused on risk and protective factors for cognitive decline, along with practical memory enhancement strategies. After completing the study, participants in the CG will be given the option to receive one of the two intervention, upon request.

#### Therapies Frameworks

Two therapeutic frameworks underpinned this study: 1) the International Classification of Functioning, Disability and Health (ICF)^62^, which was used to classify SCI and MCI as health conditions and 2) the Occupational Therapy Practice Framework, 4th edition (OTPF-4)^63^, which guided the intervention process in conjunction with the applied therapeutic models.

The ICF is a standardized and universal classification system that provides a framework and common language for describing and defining the state of health. It enables the identification of limitations and/or restrictions arising from diseases or disorders^62^.

The OTPF was developed to define the unique perspective of OT and its contribution to promoting health and participation across individuals, groups, and populations. “Achieving health, well-being, and participation in life through engagement in occupation” summarizes the domain and process of OTPF-4 as outlined in the framework^63^.

*Occupational Therapy Models.* Two occupational therapy models were used in the study:

- Model of Human Occupation (MOHO) by Gary Kielhofner^64^, which is a widely recognized framework in occupational therapy. It is occupation-centred and views the individual as a dynamic system to promote well-being and quality of life^65^^,^^66^.
- The Cognitive Model focuses on the development and study of cognitive functions based on principles of information processing. Cognitive functions emphasize problem solving as an occupational activity, structuring task performance through a hierarchy - from basic to more complex cognitive skills and abilities^67^.

#### Assessments

Trained professionals (psychologists and occupational therapists) will carry out the assessments. The evaluation protocol includes one pre-intervention assessment and follow-up assessments at Week 1, Month 6, and Month 12. In alignment with the WHO 2024 Good Practice Guidance for Clinical Trials^68^, extended follow-up allows for the identification of lasting or delayed effects of the intervention, should they occur. An evaluation protocol will be applied that included *ad hoc* variables of socio-demographic nature (clinical characteristics and lifestyle). These variables were documented in a socio-health record.

The socio-demographic variables include age, sex, marital status, educational level, physical and mental occupational status, household composition, and interests, roles, and values.

The above-mentioned variables will be categorized as follows:

- Education level: primary, secondary, and higher education.
- Occupational status (physical and mental): low, medium, and high in each case.
- Household composition: living alone, living with a partner, living with a partner and children, and living with other family members.
- Interests, roles, and values: no interests, 1 to 3 interests, and more than three interests; no role, 1 to 3 roles, more than 3 roles; none, personal, and social, respectively.

Assessed clinical characteristics will be chronic pathologies, alcoholism, and treatment for anxiety and depression.

Assessed lifestyle variables will be smoking, physical activity, and Mediterranean diet.

#### Primary Variable

The primary endpoint will be the change in cognitive performance, assessed using the MEC-35. The MEC-35 also functions as a screening instrument for MCI in cases where an official diagnosis is either unavailable due to data protection regulations or has not yet been established. The MEC-35 is the Spanish adaptation of the Mini-Mental State Examination (MMSE) that remains one of the most widely used cognitive screening tool to assess cognitive impairment in the primary care setting. It serves as a benchmark against which newer instruments are often compared^69^. Adapted by Lobo et al. for Spanish-speaking populations, the MEC-35 includes minor modifications to the original MMSE, most notably an increased total score of 35 points instead of 30^70^.

The test assesses eight cognitive domains: temporal and spatial orientation (ten items), fixation memory (three items), attention (three items), calculation (five items), short-term memory (three items), and language and praxis (11 items). Its sensitivity is 89.8% and its specificity 83.9%^57^.

#### Secondary variables

Scores in the following tools: Test for Memory Impairment (T@M), Set-Test (S-T), T-ADLQ, Lawton and Brody (L-B) scale, Yesavage Geriatric Depression Scale - 15 item version (GDS-15) and the Goldberg anxiety subscale.

- The T@M is a verbal episodic and semantic memory screening test designed to detect SCI and MCI^71^. The test has a maximum score of 50 points, with one point awarded for each correct answer. All questions are administered orally and have a single answer^71^. The T@M consists of five subtests: encoding (5 points), orientation (10 points), semantics (15 points), free recall (10 points), and guided recall (10 points)^71^. The test assesses temporal orientation and memory (episodic, textual, and semantic)^72^. In individuals aged 55 and older, a cut-off score of 46.50 yielded a sensitivity of 81% and specificity of 61% for distinguishing individuals with normal cognition from those with MCI (AUC = 0.76, p < 0.0001). A cut-off score of 45.50 showed 92% sensitivity and 73% specificity for identifying MCI (AUC = 0.88, p < 0.0001), while the same cut-off score demonstrated 63% sensitivity and 73% specificity for differentiating between individuals with SCI and those with MCI (AUC = 0.69, p < 0.0021). The T@M also shows adequate internal consistency (= 0.75) and convergent validity with the MMSE (r = 0.37, p < 0.0001)^71^.
- The S-T assesses semantic fluency across four categories: colours, animals, fruits, and cities. Scores range from 0 to 40, with 0 being the minimum score and 40 the maximum. A cut-off point below 25 points may predict progression from mild MCI to dementia^73^. The questionnaire has demonstrated a sensitivity of 79% and a specificity of 82%^74^.
- The T-ADLQ assesses seven domains of daily living: self-care (6 items), home care and management (6 items), work and recreation (4 items), shopping and money (3 items), travel (3), communication (5 items), and technology use (5 items). Each item is scored from 0 (no difficulty) to 3 (unable to perform the activity). Items not applicable to a participant’s routine are excluded from scoring. The T-ADLQ shows high internal consistency (Cronbach’s α = 0.861) and, at a functional impairment cut-off point > 29.25%, shows 82% sensitivity and 90% specificity^75^. The total scale and its dimensions (basic, instrumental, and advanced ADLs) have strong psychometric properties in individuals aged > 50 years, both cognitively healthy and healthy and impaired^76^.
- The L-B scale assesses independence in eight IADLs. Scores range from 0 (dependent) to 8 (independent). When dependences in three activities were observed, the scale has 57% sensitivity and 82% specificity^77^. It is used to assess real-life functioning in cognitively stimulating leisure activities among individuals with MCI^78^.
- The GDS-15 is used to screen for depression symptoms - this scale is considered suitable for community-dwelling younger and older adults^79^. Scores range from 0 to 15, with scores > 5 indicating *probable depression*^80^. The GDS-15 reports good diagnostic sensitivity and specificity in younger (72% and 97%) and older (86% and 91%) adults^79^. Its internal consistency is 0.812 in individuals with mild and moderate deterioration. A cut-off of 8/9 yields 90% sensitivity and 62% specificity in mild-moderate deterioration^81^.
- To assess anxiety, the Goldberg anxiety subscale will be used. It consists of nine dichotomous (yes/no) items, with one point assigned per affirmative response. The cut-off score of ≥ 4 indicates *probable anxiety*. This scale shows a specificity of 91% and a sensitivity of 86%^82^ and is validated for older adults with MCI^83^.
- In addition to the above assessments, the questionnaire *Cognitively stimulating cognitive leisure activities: scoring based on its three components at different stages of the life*, adapted from Karp et al. 2006^61^ will be administered. Reliability analyses of the three components show high internal consistency, with a Cronbach’s alpha of 0.90 overall. The specific values are 0.89 for the mental component, 0.95 for the physical component, and 0.82 for the social component.

#### Sample size

Sample size was calculated using a mixed between-within subjects repeated measures ANOVA, based on data from a study^47^ for MEC-35 in the mildly impaired group. With a calculated effect size of η^2^_partial_ = 0.864, a significance level of α<0.05, power of 85%, and assuming a 15% dropout rate, a minimum of 59 participants are needed.

### Statistical analysis

Statistical analysis will be performed using the R software, version 3.5.1. (R Foundation for Statistical Computing, Institute for Statistics and Mathematics, Welthandelsplatz 1, 1020 Vienna, Austria). Analysis will follow a per-protocol approach, without imputation for missing data. A significance level of p < 0.05 will be established.

Quantitative variables will be reported as mean ± standard deviation (SD); qualitative as absolute and relative (%) frequencies. The Shapiro-Wilk test will assess the normality of quantitative variables.

For outcome variables with non-normal distribution, a robust repeated measures model will be used - incorporating between-subject (group) and within-subject (measurement) factors - based on 20% trimmed means. Post hoc comparisons will be conducted using the Mann-Whitney U-test (between groups) and the Wilcoxon signed-rank test (within groups) with Bonferroni correction. Variables with normal distribution will be analysed using a parametric mixed ANOVA. Homogeneity of variance will be assessed with Mauchly’s test. Post hoc comparisons will be conducted using independent samples t-tests (between groups) and paired t-tests (within groups), with Bonferroni correction will be applied in both.

Effect size will be calculated using bootstrapped partial eta squared (η2p), classified as small (< 0.06), moderate (0.06-0.14), or large (> 0.14)^84^.

### Ethics committee review and approval

This study was approved by the Research Ethics Committee of the Autonomous Community of Aragon (protocol number CEICA PI23/368). All personal data protection regulations were followed. Participants were informed of the study’s objectives and provided written informed consent. The protocol complies with the ethical principles of the 1964 Declaration of Helsinki, as revised at the 64^th^ WMA General Assembly in Fortaleza, Brazil (2013)^85^, and conformed to Good Clinical Practice guidelines and applicable legislation.

## LIMITATIONS

There are some limitations to this study: 1) the use of new technologies in IG1; 2) the MCI subtype of participants is unknown; 3) the SLA categories are not standardised, which may affect the reliability of the results.

To minimise these limitations, we will adopt the following measures: 1) participants will receive training in the use of technology before and during the intervention. In face-to-face sessions, we will address doubts through hands-on practice, and participants will be offered ongoing support via telephone for home-based queries; this tutoring aims to ensure adherence to the intervention protocol; 2) although the MCI subtypes are not identified, the exercises will be adapted and personalised based on each participant’s cognitive level and the affected cognitive neurocognitive constructs; 3) SLA categories will be record weekly and converted into an ‘overall frequency’ score. These will be then classified *high*, *moderate*, or *low* engagement, based on the mental, physical, and social components of the activities.

## Data Availability

They are available upon request to the corresponding author.
